# Antimicrobial Potential of Bee-Derived Products: Insights into Honey, Propolis and Bee Venom

**DOI:** 10.3390/pathogens14080780

**Published:** 2025-08-06

**Authors:** Agnieszka Grinn-Gofroń, Maciej Kołodziejczak, Rafał Hrynkiewicz, Filip Lewandowski, Dominika Bębnowska, Cezary Adamski, Paulina Niedźwiedzka-Rystwej

**Affiliations:** 1 Institute of Biology, University of Szczecin, 71-412 Szczecin, Poland; agnieszka.grinn-gofron@usz.edu.pl (A.G.-G.); 230155@stud.usz.edu.pl (M.K.); filip.lewandowski@usz.edu.pl (F.L.); dominika.bebnowska@usz.edu.pl (D.B.); cezary.adamski@usz.edu.pl (C.A.); paulina.niedzwiedzka-rystwej@usz.edu.pl (P.N.-R.); 2 Center for Experimental Immunology and Immunobiology in Infectious Diseases and Cancer, University of Szczecin, 71-412 Szczecin, Poland; 3 Regional Centre for Digital Medicine, Pomeranian Medical University in Szczecin, 70-204 Szczecin, Poland

**Keywords:** bee products, antimicrobial resistance, apitherapy, antimicrobial activity, bees, natural bioactive substances, antibacterial, antiviral, antifungal

## Abstract

Bee products, in particular honey, propolis and bee venom, are of growing scientific interest due to their broad spectrum of antimicrobial activity. In the face of increasing antibiotic resistance and the limitations of conventional therapies, natural bee-derived substances offer a promising alternative or support for the treatment of infections. This paper summarizes the current state of knowledge on the chemical composition, biological properties and antimicrobial activity of key bee products. The main mechanisms of action of honey, propolis and bee venom are presented, and their potential applications in the prevention and treatment of bacterial, viral and fungal infections are discussed. Data on their synergy with conventional drugs and prospects for use in medicine and pharmacology are also included. The available findings suggest that, with appropriate standardization and further preclinical and clinical analyses, bee products could become an effective support for the treatment of infections, especially those caused by pathogens resistant to standard therapies.

## 1. Introduction

Honey bees, belonging to the order of Hymenoptera, suborder Apocrita and family Apidae, are insects that play a fundamental role in shaping the balance of ecosystems, contributing to the pollination of a significant part of crop plants around the world [1,2,3,4]. According to estimates, almost 70% of global crop production is dependent on the activity of pollinators, primarily bees, whose functioning ensures stability and biodiversity [2,3,5]. From the perspective of humans, and especially agriculture, the role of bees translates into real economic benefits, which amount to many billions of euros per year. It is estimated that honeybees contribute directly to global agriculture to the amount of about EUR 153 billion per year [1,6]. Unfortunately, in recent decades there has been a steady decline in the numbers of these insects, caused by the impact of numerous biotic (parasites, infectious diseases) and abiotic (climate change, pesticides) factors, which poses a serious challenge to global food security [2,3,6,7].

Bees, in addition to their key role in pollinating plants, are also valued for providing products with high therapeutic potential. Of particular importance among them are honeybees, such as *Apis mellifera* (found mainly in Europe, America, Africa and Asia) and *Apis cerana* (from Southeast Asia), which produce a wide range of bee products rich in bioactive compounds, including polyphenols, flavonoids, proteins, organic acids and enzymes. The most important of these include honey, bee pollen, bee bread, royal jelly, propolis and bee venom, valued for centuries for their potential health-promoting properties [8,9,10,11,12,13,14]. In the traditions of many cultures, these products have held an important place in phytotherapy for centuries, which is confirmed by historical references to their use in healing wounds, treating skin diseases and digestive system diseases [15,16]. An example would be the use of honey in ancient Egypt to disinfect wounds or propolis in ancient Greece as an antiseptic substance [17,18]. Nowadays, thanks to the development of biological and medical sciences, we are increasingly understanding the mechanisms of action of these natural products, and numerous studies indicate their antimicrobial, anti-inflammatory, immunomodulatory and antioxidant properties [3,5,19,20,21].

The importance of the biological properties of bee products is particularly important in the face of the dynamic increase in infections of various etiological origins (bacterial, viral, fungal) [3,5,19,20,21]. In the era of intensive antibiotic therapy and progressive multidrug resistance of pathogens, the search for new, effective and safe treatment assays is a priority for scientists and medical practitioners. An additional challenge is the toxicity of many commonly used synthetic drugs and limitations in their long-term use, which encourages the development of alternative or supportive therapies, including those based on natural substances [22]. The multifaceted effects of bee products make them a promising component in the fight against respiratory and digestive system infections, as well as in the prevention of skin diseases and oral diseases [3,5,19,20,21].

This article aims to discuss and systematize knowledge about the therapeutic potential of bee products in the treatment and prevention of infections of various etiologies. In particular, the mechanisms of action of honey, propolis, pollen, bee bread, royal jelly and venom will be presented, as well as the prospects for their use in combination with conventional pharmacotherapy. Analysis of available studies, while indicating significant limitations and potential risks, will allow for a broader understanding of how bee products can support efforts to combat increasingly frequent and complex health problems associated with infections. In the context of global challenges, such as multidrug resistance of microorganisms or limited access to modern drugs, further research and development of apitherapy is indicated as an important complement to standard medical care.

## 2. Bee Products and Their Chemical Composition

Bee products play a crucial role in the functioning of the hive, fulfilling a variety of biological and ecological functions [23,24,25]. Honey serves as the primary food source for bees, providing essential carbohydrates [26], while propolis, also known as “bee glue”, is used to seal gaps and protect the colony from pathogens [27]. Bee venom functions as a defense mechanism against predators [28]. The production processes of these substances are complex and depend on the interactions between bees and their environment [23,24,25,26,27,28]. Honey is produced by collecting nectar from flowers, which is then processed and stored in honeycombs [23]. Propolis is gathered by bees from plant resins and subsequently modified through the addition of waxes and enzymes [29]. Bee venom is synthesized in the venom glands of worker bees and deployed in threatening situations [30].

Beyond their medicinal uses, bee products are widely applied in various fields [31,32,33,34]. Honey is valued in cuisine for its flavor and nutritional properties [32], propolis is used in cosmetics due to its antiseptic qualities [33], and beeswax finds application in both industry and craftsmanship [35].

From a chemical perspective, bee products are rich in a wide range of bioactive compounds [36,37,38,39]. Honey consists primarily of simple sugars, but also contains proteins, enzymes, amino acids, minerals, vitamins, and polyphenols [11]. Propolis is a source of flavonoids, phenols, terpenes, and other compounds with antioxidant and antibacterial effects [40,41]. Bee venom contains peptides such as melittin and apamin, as well as enzymes including phospholipase A_2_, all of which exhibit diverse biological activities [42]. In scientific research, bee products are often used in various formulations [43,44].

Ethanolic and aqueous extracts are commonly employed to isolate bioactive compounds from propolis [43], while supercritical CO_2_ extraction allows for the acquisition of specific, highly pure fractions [45]. Vacuum and freeze-drying honey [46] and purified peptides from bee venom are also used in studies investigating their biological properties [47]. Standardization of these preparations is essential to ensure the reproducibility of results and the reliability of research [44,48].

### 2.1. Honey

Honey is a natural product produced by bees from plant-derived raw materials. Honey bees (*Apis mellifera*) collect nectar or honeydew (sweet secretions of sap-sucking insects) from plants and transform it into mature honey within the hive. Floral nectar typically contains 40–80% water and a significant amount of sucrose, whereas honeydew has a slightly different sugar composition—often richer in complex sugars (e.g., melezitose, raffinose) and with a higher mineral content [36,49]. For this reason, honeydew honey (originating, among others, from coniferous trees such as fir and spruce, as well as from deciduous trees like oaks and lindens) differs from nectar honey in color (it is darker), composition, and health-promoting properties [36,37]. Honeydew honey contains on average about 1% mineral components, whereas typical nectar honey contains only 0.1–0.5% [49]. Furthermore, honeydew honeys are found to contain more oligosaccharides and dextrins, whereas nectar honeys are dominated by simple sugars (glucose and fructose) and disaccharides [38,39]. However, the production process of both honey types is similar—bees concentrate the raw material in the hive and enrich it with enzymes, regardless of whether the source is nectar or honeydew [37,40].

Freshly collected nectar or honeydew is transported in the bee’s honey stomach (the so-called “honey crop”) and undergoes initial enzymatic processing during transport. Forager bees repeatedly detach and pass nectar droplets between one another, adding enzymes and evaporating water in the process [41]. Subsequently, hive bees place the processed liquid into honeycomb cells and accelerate further evaporation of water by vigorously fanning their wings. When the water content drops from approximately 60–80% to around 17–20%, and the sugar concentration reaches saturation, the bees seal the honeycomb cells with wax, protecting the mature honey from absorbing moisture and spoiling [50]. The final product—mature honey—takes the form of a thick, viscous liquid with high sweetness, capable of long-term storage due to its low water content and unique composition [51].

#### 2.1.1. Enzymes of Honey

Enzymes added by bees play a key role in processing plant-derived raw materials into honey [52,53,54,55]. These enzymes originate primarily from the bees’ salivary glands (mainly the hypopharyngeal glands), and partly also from the plants themselves (nectar) and microorganisms present in the raw material [52]. The most important enzyme is invertase (sucrase)—secreted by the bees during nectar transport—which catalyzes the hydrolysis of sucrose (the dominant sugar in nectar) into two simple sugars: glucose and fructose [53]. As a result, honey mainly contains easily digestible simple sugars, with almost all sucrose being broken down (in ripe honey, the sucrose content is below ~5%) [54]. Another enzyme introduced by bees is α- and β-amylase (diastase), which breaks down any polysaccharides (e.g., starch from pollen grains or honeydew) into dextrins and simpler sugars [55]. Diastase activity is a traditional indicator of honey quality and freshness—this enzyme is quite sensitive to heating and prolonged storage, so its level decreases in overheated or old honey [52].

Another key enzyme is glucose oxidase (GOX), also added by bees during the maturation of honey [56]. This enzyme oxidizes a portion of glucose into gluconic acid and hydrogen peroxide (H_2_O_2_) in the presence of oxygen and the FAD cofactor [57]. Gluconic acid lowers the pH of honey (typically to ~3.2–4.5), giving it its characteristic acidity, while hydrogen peroxide is responsible for honey’s so-called “peroxide activity”—its antiseptic and bactericidal properties [58] In fresh, mature honey, the concentration of H_2_O_2_ is kept at a safe level thanks to the activity of catalase—an enzyme that breaks hydrogen peroxide down into water and oxygen [52]. Interestingly, catalase enters honey primarily from nectar and pollen, as well as from microorganisms, rather than from the bees themselves [56]. In honeys with high catalase activity (e.g., some honeydew honeys or manuka honey), very low levels of H_2_O_2_ are observed, whereas honeys with low catalase activity accumulate more peroxide and display stronger bactericidal peroxide activity [52]. In addition to the aforementioned enzymes, honey also contains others such as phosphatases, lysozyme, β-glucosidase, and plant-derived enzymes, which may continue to modify honey’s composition during maturation [59]. Thanks to the synergistic action of enzymes and the evaporation of water, raw nectar or honeydew is transformed into stable honey, rich in simple sugars, with an acidic pH and the presence of bioactive components [52].

#### 2.1.2. Chemical Composition of Honey

Honey is a mixture of several hundred different chemical compounds, with carbohydrates being the dominant component [32,36,49,60]. On average, the total sugar content in mature honey ranges from 70 to 85% by weight, with the main constituents being monosaccharides: fructose (approximately 30–45%) and glucose (approximately 25–40%) [49]. The fructose-to-glucose ratio is usually >1 (typically in the range of ~1.2–1.7), which makes honey slightly sweeter than sucrose—fructose has a higher sweetness level than glucose [60]. In addition to simple sugars, honey also contains disaccharides (up to around 5–10%, mainly maltose, isomaltose, sucrose, and trehalose), as well as oligosaccharides and dextrins (together up to a few percent) [49]. The exact sugar profile depends on the origin of the honey—honeydew honeys contain more complex sugars (e.g., melezitose, which can crystallize into so-called “cement honey”), whereas nectar honeys are dominated by glucose and fructose [32,36].

The second most important component of honey is water—its content typically ranges from 16 to 20% [61]. The low water activity (aw ~0.5–0.6) is responsible for honey’s long shelf life and its ability to inhibit microbial growth [62]. If the water content exceeds 20%, honey becomes susceptible to fermentation by osmotolerant yeasts, which is why bees carefully regulate this parameter during the maturation process [61,62].

Although sugars and water account for over 90% of honey’s mass [36,49,60,61,62], the remaining small fraction (5–10%) is responsible for many of its unique biological properties [49,52,63,64]. Honey contains organic acids (0.5–1%), primarily gluconic acid (a product of glucose oxidase activity), as well as malic, citric, succinic, and other acids—these are what give honey its acidity (pH ~3.5) [52,64]. Proteins and nitrogenous compounds make up approximately 0.2–0.5% of honey—these include enzymes (invertase, diastase, glucosidase, catalase, phosphatase, and others), free amino acids (over 25 have been identified, with proline—mainly from bee secretions—being the most abundant), and trace amounts of proteins derived from pollen [49]. The content of free amino acids and proteins also affects honey’s tendency to brown (Maillard reactions) during aging and heating [64].

Honey contains small amounts of vitamins, primarily from the B group, including thiamine (B_1_), riboflavin (B_2_), niacin (PP), pyridoxine (B_6_), pantothenic acid (B_5_), biotin (H), and folic acid (B_9_), as well as vitamins C, K, and trace amounts of vitamin A [49,65,66]. However, the overall vitamin content is low—typically several dozen milligrams per kilogram of honey—making it an insignificant dietary source of vitamins [65].

Of much greater importance for honey’s biological activity are its phenolic compounds and flavonoids. Although present only in trace concentrations (tenths or hundredths of a percent), these compounds exhibit notable antioxidant, anti-inflammatory, and antimicrobial effects [49,67,68]. The phenolic profile of honey varies with its botanical origin—for instance, buckwheat honey contains rutin and gallic acid, manuka honey is rich in leptosperin and methylglyoxal, while eucalyptus honeys contain ellagic acid [36]. These phenolic acids and flavonoids are regarded as the main contributors to honey’s antioxidant potential and, to a lesser extent, its antimicrobial properties [49,63]. Darker honeys (e.g., buckwheat, honeydew, or heather honey) typically contain higher levels of phen chrysin olics and show stronger antioxidant and antibacterial activities compared to lighter honeys such as acacia [36]. Notably, several flavonoids commonly identified in honey, such as apigenin, chrysin, pinocembrin, eupatilin, myricetin, quercetin and phloretin, have been shown to exert a broad spectrum of biological activities. These include anti-inflammatory, antioxidant, photoprotective, antimicrobial and even anticancer properties, particularly in relation to skin health. Their presence, even at low concentrations, reinforces the therapeutic potential of honey and supports its application beyond simple nutrition [11,69].

Honey also contains approximately 20–30 mineral elements [49,70,71]. Potassium (K) is the predominant mineral, accounting for a significant portion of the ash content. Other minerals include calcium, sodium, magnesium, phosphorus, iron, zinc, manganese, silicon, and various trace elements [70]. The total ash content ranges from 0.1 to 0.5% in nectar honeys and can reach up to 1% in honeydew honeys [49]. Mineral composition is strongly influenced by the soil conditions where nectar-producing plants grow, which is why it can serve as an indicator of honey’s geographical origin [71]. Although the overall mineral percentage is low, these elements are important for the electrical conductivity of honey (used in the identification of honeydew honeys) and may influence enzymatic activity and product stability [49,70,71].

#### 2.1.3. Forms of Honey Utilized in Research

In laboratory studies on the antimicrobial properties of honey, various forms and preparations of the product are used, with efforts made to ensure proper standardization [72,73,74,75,76,77]. The simplest form is raw honey—taken directly from the comb (after centrifugation and, if necessary, filtration to remove wax) [72]. This type of honey is used, for example, in vitro tests as a complete research sample [72]. It is often dissolved in sterile water or broth to a defined concentration in order to determine the minimum inhibitory concentration (MIC) or zones of growth inhibition on solid media [49,70,71]. Typical concentrations of honey tested against bacteria range from 5 to 50% (v/v) [75]. Due to its high viscosity and sugar content, it is often necessary to compare the effects with so-called artificial honey—a sugar solution that mimics the sugar composition of natural honey but lacks biologically active components. This allows researchers to determine what portion of the antimicrobial effect is due to osmotic pressure (sugars) and what portion results from honey’s unique bioactive compounds [55]. For example, Mundo et al. demonstrated that artificial honey (a mixture of glucose, fructose, and maltose in concentrations corresponding to natural honey) inhibits bacterial growth significantly less effectively than real honey, confirming the contribution of hydrogen peroxide and other compounds to its antibacterial activity [77].

Recent studies have also demonstrated that specific phenolic compounds present in honey—such as p-coumaric acid, hydroxyphenyl acetic acid, 1H-quinolinone, and abscisic acid—strongly correlate with antimicrobial activity. These molecules may act via multiple mechanisms, including membrane disruption, interference with bacterial DNA replication, or modulation of gene expression. For example, p-coumaric acid, abundant in buckwheat honey, has been shown to inhibit the growth of *S. aureus* and *E. coli*. This highlights the importance of not only total phenolic content, but also specific compound composition when evaluating the therapeutic potential of honey [78].

### 2.2. Propolis

Propolis (bee glue) is a resinous substance collected by bees from plant buds and exudates, which they then use inside the hive to seal gaps and smooth the surfaces of the nest [79]. Thanks to its antimicrobial properties, propolis forms a protective barrier within the hive that limits the growth of bacteria and fungi, helping to maintain a healthy microclimate for the colony [80]. This phenomenon is considered part of the so-called social immunity of honeybee colonies [79]. Bees are also capable of covering the bodies of dead intruders that are too large to remove from the hive with a layer of propolis, effectively mummifying them and preventing the decomposition of their remains [79,81].

Raw propolis typically contains approximately ~50% plant-derived resinous substances, ~30% beeswax, ~10% essential oils, with the remainder made up of pollen and other impurities [29,79,82]. In total, more than 300 different chemical compounds have been identified in propolis [63,64]. The main groups include polyphenols, primarily flavonoids, phenolic acids and their esters, and terpenes [83]. Propolis also contains waxes and aromatic essential oils [79,83,84]. Among the aromatic acids found in propolis are caffeic acid, ferulic acid, and cinnamic acid [85].

The composition of propolis depends on the source of resins collected by bees, which varies according to local flora and geographical region [84,86,87,88]. In temperate climates (e.g., in Europe), bees mainly gather propolis from the buds of poplar trees (Populus spp.), which is why European propolis (typically brown) is rich in flavonoids and other polyphenols characteristic of poplar exudates [29]. In contrast, Brazil is known for its so-called “green” propolis, derived from the plant *Baccharis dracunculifolia*, which is distinguished by a high content of prenylated derivatives of cinnamic acid (e.g., artepillin C) [89]. Meanwhile, “red propolis”, found for instance in Cuba, is produced from the resins of plants such as *Clusia* spp., and contains characteristic polyprenylated benzophenones not found in temperate-zone propolis [90].

Propolis shows strong antimicrobial activity. Numerous studies have confirmed the efficacy of this natural product against both Gram-positive and Gram-negative bacteria, viruses, as well as pathogenic fungi [91,92]. It also possesses high antioxidant potential, owing to the presence of numerous polyphenolic compounds capable of neutralizing free radicals [85], propolis has well-documented anti-inflammatory properties, contributing to the inhibition of inflammatory mediators and modulation of the body’s immune response [93].

In scientific research on propolis, the most commonly used preparation is the ethanolic extract of propolis (EEP), which effectively extracts biologically active components [79]. Alternatively, non-alcoholic extracts, such as glycerine-based ones, are also used, although they differ in composition and activity from EEP [94]. To analyze the individual components of propolis, researchers also isolate phenolic fractions or specific pure compounds, for example, caffeic acid phenethyl ester (CAPE), to study their individual biological effects [95].

### 2.3. Bee Venom

Bee venom (apitoxin) plays a key role within the bee colony, primarily as a defensive agent protecting the hive from intruders [30,96,97]. Worker honeybees possess stingers connected to venom glands, and when threatened, they inject a dose of venom into the target—an effective defense mechanism [30]. Components of the venom (e.g., melittin) cause an immediate, sharp pain in the attacked organism [97], which deters predators and intruders from continuing their assault [30]. There are also reports suggesting that venom may play an additional role in maintaining colony health, antimicrobial peptides derived from bee venom have been detected on wax combs and bee bodies, suggesting a possible contribution of venom to bee social immunity [96].

Chemically, bee venom is a complex mixture of biologically active compounds. Over 80% of its mass is water, while the remainder consists of a diverse range of components, including proteins (enzymes), peptides, biogenic amines, and other substances [30,42,98]. The main component of bee venom is the peptide melittin, which makes up approximately 50–60% of its dry mass [42]. Other peptides are also present, such as apamin (~2%), a neurotoxin that blocks Ca^2+^ —dependent potassium channels in nerve cells, and the mast cell degranulating peptide (~2%), which has anti-inflammatory properties [98]. Important enzymatic components include phospholipase A_2_ (approximately 10–12%) [42] and hyaluronidase (approximately 1–3%) [42]. Phospholipase A_2_ hydrolyses phospholipids in cell membranes and, in combination with melittin, causes significant membrane damage [99], while hyaluronidase degrades hyaluronic acid in host tissues, increasing their permeability and facilitating the spread of venom [100]. Both enzymes are considered among the most potent allergens in bee venom [30]. Bee venom also contains small amounts of biogenic amines such as histamine and catecholamines (dopamine and noradrenaline) [101]. Histamine increases capillary permeability and intensifies the inflammatory response, whereas catecholamines raise blood pressure and accelerate heart rate, which helps to distribute the venom more rapidly throughout the victim’s body [30].

Melittin, the main component of bee venom, is a polypeptide made up of 26 amino acids, positively charged and amphipathic in nature [102]. By binding to the lipids of cell membranes, melittin forms pores (~4 nm in diameter), leading to cell lysis [103]. As a result, melittin exhibits strong cytolytic activity, causing, among other effects, haemolysis (the destruction of red blood cells) due to membrane damage [104]. Numerous preclinical studies have also confirmed its anticancer properties—melittin inhibits growth and induces death in cancer cells across various models (including melanoma, lung cancer, glioma, and leukemia) [102,105,106,107,108]. Cancer cells have been observed to be more sensitive to melittin than healthy cells, suggesting a degree of selectivity in its action [102]. The mechanisms underlying its anticancer effect include the induction of apoptosis and disruption of key signaling pathways that regulate cancer cell survival and proliferation [109]. In addition, melittin displays immunomodulatory properties—for instance, it inhibits NF-κB activity and reduces the release of pro-inflammatory mediators (e.g., TNF-α), thereby helping to alleviate inflammatory responses [110,111].

Research on bee venom faces several significant challenges. One of them is obtaining sufficient quantities of the raw material with standardized quality. The most commonly used assay involves electrically stimulating bees to sting a special collection membrane [112]. This technique avoids killing the bees but may result in the loss of certain volatile components (e.g., histamine) during collection [30]. An alternative approach is the extraction of venom directly from the venom gland; however, material obtained this way is often contaminated with fragments of bee tissue and tends to be of lower purity [113]. Another major challenge is the risk of severe allergic reactions [30]. Bee stings are among the most common causes of anaphylaxis—it is estimated that approximately 8% of the population experiences anaphylactic shock following a sting [104]. The main allergen in bee venom is phospholipase A_2_, but melittin and hyaluronidase also possess strong allergenic properties [114]. The risk of a sudden allergic reaction is a serious limitation to the potential therapeutic use of bee venom [30,114].

## 3. Antimicrobial Activity of Bee Products

### 3.1. Characterization of Microbial Infection

Human infections are caused by a diverse array of bacterial and fungal pathogens, which collectively exact a massive toll on global health. Bacterial infections alone were associated with an estimated 7.7 million deaths in 2019—roughly one in eight of all deaths worldwide. A recent systematic analysis identified 33 bacterial species or genera as the predominant causes of these deaths [115]. Among these, five pathogens—*Staphylococcus aureus*, *Escherichia coli*, *Streptococcus pneumoniae*, *Klebsiella pneumoniae*, and *Pseudomonas aeruginosa*—accounted for about 55% of bacterial infection fatalities. These exemplify the major taxonomic groups of bacterial pathogens: Gram-positive cocci and Gram-negative rods are common causes of human disease. Other important groups include the Mycobacteria; notably Mycobacterium tuberculosis remains a leading single-agent killer, causing ~1.4 million deaths in 2021, with especially high burdens in low-resource regions [116]. In 2019, over 6 million deaths were attributed to just three syndromes caused by bacteria, reflecting the enormous number of severe bacterial infections occurring globally each year [115].

Fungal pathogens, also contribute substantially to human morbidity and mortality. Fungi are ubiquitous in the environment and an estimated 3–5 million species are able to cause disease in humans [117]. The pathogenic fungi belong primarily to the phyla Ascomycota and Basidiomycota, and species from four genera—*Aspergillus*, *Candida*, *Cryptococcus*, and *Pneumocystis*. These fungi cause the majority of life-threatening invasive fungal infections. For example, Candida yeasts (especially *C. albicans* and related species) are the most common cause of invasive mycoses such as candidemia, while *Aspergillus fumigatus* and relatives cause invasive aspergillosis in immunosuppressed hosts [118]. *Cryptococcus neoformans* causes *cryptococcal meningitis*, and *Pneumocystis jirovecii* causes pneumonia in immunocompromised people. An estimated >150 million people develop serious fungal diseases annually. Global deaths due to fungal infections are now estimated at approximately 1.5–1.6 million per year [117].

Both bacteria and fungi are increasingly developing resistance to antimicrobial therapies, undermining our ability to treat infections. Antimicrobial resistance (AMR) in bacteria has reached crisis levels globally. In 2019, an estimated 4.95 million deaths were associated with drug-resistant bacterial infections, including 1.27 million deaths directly attributable to AMR [119]. Common pathogens that were once readily treatable now possess strains resistant to multiple antibiotic classes. For instance, methicillin-resistant *S. aureus* (MRSA) has spread worldwide in hospitals and communities [120] and was responsible for over 100,000 deaths in 2019. Similarly, multi-drug-resistant *E. coli* and *K. pneumoniae*, *Acinetobacter baumannii* resistant to nearly all β-lactams, and fluoroquinolone-resistant *P. aeruginosa* are causing difficult to treat infections. These six leading drug-resistant bacteria together accounted for about 930,000 deaths attributable to AMR in 2019 [119]. These trends underscore that decades of antibiotic usage have accelerated the evolution of resistance [121]. Bacteria acquire resistance genes via mutations or horizontal gene transfer, enabling them to neutralize drugs, alter drug targets or expel drugs. The result is that infections which were once routine to cure can become resistant, necessitating last-line therapies or leaving no effective treatment [122]. Without concerted action, the spread of multidrug-resistant bacteria threatens to reverse the success of combating microbial infections the availability of antibiotic treatment allowed.

### 3.2. Antimicrobial Activity of Honey

The antimicrobial activity of honey has been recognized since the late XIX century [123]. Modern research has shown it exhibits significant activity against many bacteria and other microorganism species. Due to the difference in chemical composition based on the nectar or honeydew source [124] the effectiveness of local honeys can significantly differ between each other.

It has been established that honey’s antibacterial effect is the result of two pathways. The peroxide dependent pathway is the result of the presence of glucose oxidase in honey. This enzyme, by catalyzing the oxidation of glucose to β-gluconolactone, produces hydrogen peroxide—H_2_O_2_ [125], which has a directly bactericidal effect, causing oxidative damage to cell structures [126], and degradation of DNA [127]. It has been show that this mechanism is the major contributor to the antibacterial effect, with the notable exception of manuka honey [128]. The rest of the antibacterial effect of honey is ascribed to the peroxide independent pathway, which is the result of various other physiochemical properties of the substance. The high sugar content resulting in high osmolality causes cellular dehydration due to osmotic pressure [72]. The typical pH of honey, 3.2–4.5 [63], is outside the range well tolerated by most common bacterial pathogens [129].

Proteins other than glucose oxidase may also play a significant role, the main royal jelly protein 2 and defensin 1, both present in honey, have significant antimicrobial activity [130,131]. Phenolic, as well as flavonoid content of honey has also been correlated to its effects against bacteria [132,133]. It has to be noted that due to the range of possible chemical compositions of different honeys, there exists significant variance in the contributions of these compounds to the general effect [134]. One example of it is methylglyoxal—a bioactive compound found in Manuka honey, which has been of particular interest to researchers due to its antioxidant and antibacterial properties [135,136]. The honey is produced from nectar of *Leptospermum scoparium*, containing dihydroxyacetone [137] which is non-enzymatically converted to methylglyoxal during the honey’s maturation process. This compound has been shown to be the main cause of the Manuka honey’s non-peroxide antibacterial activity exceeding that of other products of this type [134,138].

Honey also has been shown to exhibit significant antifungal activity [134]. While the antifungal mechanisms are comparatively less researched compared to the antibacterial, it is understood that both peroxide dependent and independent pathways play a role [139]. In addition, the flavonoid content has been shown to contribute to the effect, resulting in significant research utilizing the flavonoid extract of honey against *Candida* spp. and other fungal pathogens [140].

It must be noted that honey in its typical form is limited to topical treatment, hence the need to utilize extracts, potentially expanding the uses of the substance to other routes of administration. In topical treatment, honey and honey based product has been particularly researched for wound treatment [141], as it exhibits significant activity against common pathogens in wound infection [142] (Table 1).

### 3.3. Antimicrobial Activity of Propolis

In recent years, propolis has been the subject of extensive into its potential role in medicine from many perspectives, with its antimicrobial activity being one shown a particular interest [157] (Table 2). The analysis of that property has its challenges—the form of propolis most often used is an ethanol extract, requiring higher scrutiny towards the results due to bactericidal effect of the solvent itself. This can be mitigated by utilizing DMSO in sub-inhibitory concentration as a solvent after evaporating the extract as described by Wieczyńska et al. [158]. The extract has also been shown to exhibit irregular diffusion into agar mediums, as shown by Bosio et al. [159], causing, in particular, the results of disc diffusion assays to be potentially unreliable.

Despite these limitations, it has been shown that propolis has both a direct and indirect mechanism of antibacterial activity. Propolis composition varies significantly geographically, a wide array of its biological properties is accredited to its phenolic and flavonoid content [160,161], but as described by Bouchelaghem et al. [162] a direct correlation cannot be assumed in all cases. Artepillin C, one of the antioxidant phenolic compounds whose high content is a defining characteristic of Brazilian green propolis, is a significant contributor to its bacteriostatic effect [163,164]. Prenylflavonoid constituents of propolis found in Australia [165], Taiwan and Japan [166], propolins present a significant contribution to the inhibitory activity against Gram-positive strains of bacteria. Different propolins found in the same propolis sample exhibit synergistic and inhibitory interaction towards each other’s antibacterial activity [92]. Other flavonoids also present significant activity against bacteria, apigenin [36] and pinocembrin [167] isolated from Chilean propolis exhibited ability to not only effectively inhibit growth of *S*. *mutans*, but also completely prevent biofilm formation [168]. Caffeic acid phenethyl ester (CAPE) is a compound found commonly in propolis [169], capable of inhibiting growth of *S*. *aureus*, *B*. *subtilis* and *P*. *aeruginosa* [170]. In Australian propolis, the inhibitory effect against *S. aureus* has been ascribed to C-geranyl flavonoids and triterpenoids ability to interact with the cell wall of Gram-positive bacteria [165].

Propolis has also been shown to exhibit significant antifungal properties, in particular against yeast and filamentous fungi capable of causing human infection. One of the primary mechanisms of antifungal activity has been determined to be disruption of fungal cell membrane by binding to membrane sterols [171], inhibition of cell wall synthesis by CAPE [172], and disruption of the cell’s redox balance by polyphenols, causing oxidative stress induction [173].

Due to the dynamic increase in infections with multidrug resistant bacteria [119], the perspective of utilizing propolis against bacterial strains such as methicillin resistant *S*. *aureus* has been of particular interest to researchers. Propolis has been shown to be an effective inhibitor of staphylococci growth and one not affected by antibiotic resistance mechanisms of pathogens [174,175]. Additionally, propolis exhibits ability to disrupt biofilm formation [176], downregulate expression of virulence related genes [177]. While the mechanism of antibacterial activity against *S. aureus* is not yet fully understood, it has been shown that propolis induces changes in cell wall and membrane structure of the bacteria [177]. This mechanism is also a potential explanation for another useful phenomenon—some propolis samples have been shown to have strong synergistic interactions with many antibiotics [178], in particular ones inhibiting cell wall synthesis [175], such as β-lactams and glycopeptides. Australian propolis has been found to reduce expression of genes responsible for β-lactam resistance in MRSA [81].

**Table 2:** Selection of articles on the antimicrobial effect of propolis.

Material	Microorganism	Assay	Key Results	Reference
Ethanol extract of Brazilian propolis	Clinical isolates: 210 of *S. aureus*, 48 of MRSA and 162 of MSSA	In vitro-agar dilution assay	The MIC50 and MIC 90 remained similar for all analyzed strains. Both MSSA and MRSA ATCC strains being inhibited by EEP at 1420 µg/mL concentration, showing that the mechanism of resistance to methicillin does not affect the antimicrobial effect of propolis against *S. aureus*.	[174]
Ethanol extract of Polish propolis	*S. epidermidis* strains isolated from blood samples and ATCC 35983	In vitro-Tissue culture plate assay, broth dilution assay	The extract exhibited significant antibacterial effect against *S. epidermidis*. EEP reduced bacterial biofilm formation at concentrations above 1/8 MIC, while concentrations lower than 0.025 mg/mL increased biofilm formation.	[179]
Ethanol extracts of propolis from Yangpyeong, Boryung, Cheorwon and Yeosu	*S. aureus* ATCC 25923, *B. subtilis* ATCC 15523, *S. typhimurium* ATCC 13311 *C. albicans* ATCC 10231	In vitro-disc diffusion assay, induced lipoperoxidation	Comparison of inhibition zones has shown the Yeosu and Cheorwon propolis extracts to have the strongest antimicrobial effect. These samples contained highest total polyphenol and flavonoid content and antioxidant activity.	[164]
Italian propolis dry extract dissolved in broth with DMSO and Tween 80	Clinical isolates from respiratory tract infections: *S. aureus*, β-hemolytic streptococci, *S. pneumoniae*, *M. catarrhalis*, *H. influenzae*, *K. pneumoniae*, *E. coli*, *P. mirabilis*, *P. aeruginosa* and *C. albicans* strains	In vitro-broth microdilution assay.	MIC values show propolis as an effective agent against most tested strains, except for Enterobacteriaceae, for which inhibitory effect was only achieved at high concentrations. MIC of propolis against *S. pneumoniae*, M catarrhalis and *H. influenzae* strains is within range of the respective MBC values. Bactericidal effect was shown against all isolates at 4xMIC concentration, except *S*. *pyogenes*.	[180]
Ethanol extracts of Turkish propolis from different areas of Marmara region	Clinical isolates: *E. coli*, *P. aeruginosa*, *S. aureus*, beta-hemolytic streptococci	In vitro-agar dilution assay	Analyzed samples’ MIC values showed significant difference in antibacterial effect between samples, especially against Gram-negative bacteria. The sample with the stronger antibacterial effect contained 3 chemical components not found in the less effective sample: 3-methyl-2 butenol, diethyl succinate and phenyl-ethyl alcohol.	[181]
Ethanol extracts of propolis samples from different regions of Turkey	*S. Enteritidis* ATC 13076 and *L. monocytogenes* ATCC 1462	In vitro-broth microdilution assay	All samples showed strong antibacterial effect on both species at 1:10 dilution, no viable bacteria were determined after incubation. Against *L. monocytogenes*, at 1:100 dilution 8 samples had a bactericidal effect, 11 an inhibitory effect and 6 no effect. Against *S*. *enteritidis*, 5 samples had a weak inhibitory effect and 20 no effect.	[182]
Methanol extract of Chinese red propolis	*S. aureus* ATC 25923 (methicillin sensitive), ATC 43300 (methicillin resistant)	In vitro-agar diffusion assay, broth microdilution assay, intracellular protein and nucleic acid leakage assay, metabolomic analysis,	Extract showed significant antibacterial effect against MSSA and MRSA, disrupting the cell wall, cell membrane and inducing changes in cell morphology. Metabolomic analysis showed enrichment of 12 pathways in MSSA and 9 in MRSA after treatment with the extract. Expression of genes related to biofilm formation, autolysis, cell wall synthesis and virulence of MRSA was found to be downregulated.	[177]
Ethanol, methanol, DME and aqueous extracts of Taiwanese green propolis	*S. aureus* BCRC 10780, BCRC 10781, BCRC 101451, methicillin resistant *S. aureus* ATCC 43300, *B. subtilis* BCRC 10675, *L. monocytogenes* BCRC 14845, *E. coli* BCRC 10675, *P. aeruginosa* BCRC 10944, *P. larvae* BCRC 14187	In vitro-microdilution assay	Comparable levels of antibacterial activity were exhibited by all extracts apart from the aqueous, which was unable to inhibit growth. None of the extracts inhibited growth of *E. coli*. Propolin C exhibited the lowest MIC value against Gram-positive strains. None of the tested propolis samples and propolin isolates inhibited growth of *E. coli* or *P. aeruginosa*. Out of the tested propolin combinations, twofold concentration of propolin C with propolin D exhibited highest antibacterial activity, higher than pure propolin C or total propolis extract.	[92]
Methanol extracts of Chilean propolis from the Región del Maule	*S. aureus* ATCC 25923, methicillin-resistant *S. aureus* ATCC 43300, *E. coli* ATCC 25922 and 3 clinically isolated strains, clinically isolated stains of *S. enteritidis*, *Salmonella* spp., *Y. enterocolitica*, *Pseudomonas* spp. and *P. mirabilis*.	In vitro-broth microdilution assay	Samples showed significant variance in antibacterial activity beyond the expected effect of total phenolic and flavonoid content. The highest level of activity was exhibited by central valley propolis samples. The strains most susceptible to the activity of propolis extracts were *E. coli*, *Y. enterocolitica* and *S*. *enteritidis*.	[183]
Ethanol extract of Italian propolis and bud poplar resins	*P. aeruginosa* P1232 expressing the luciferase gene and *P. aeruginosa* PAO1	In vitro-broth microdilution assay, static biofilm assay, swimming motility, swarming motility and twitching motility analysis	Both extracts exhibited comparable levels of bacteriostatic activity. At sub-MIC concentration both extracts inhibited biofilm formation and swimming motility. Bud poplar resin sample increased swarming motility, while neither sample affected twitching motility of the bacteria.	[184]
Ethanol, n-hexane, ethyl acetate and n-butanol extracts of Pacific propolis from the Guadalcanal Province	clinical isolates of methicillin resistant *S. aureus*, methicillin sensitive *S. aureus* ATC 9144, *P. aeruginosa* ATCC 25668	In vitro-agar dilution assay	Ethanol extracts exhibited the strongest antibacterial activity, showing bacteriostatic effect against all tested MRSA and MSSA strains. No samples inhibited the growth of *P. aeruginosa*. Four prenylflavanones were reported in Solomon Island propolis for the first time, propolins C and D exhibiting strong anti-MRSA activity.	[185]
Polyphenol-rich extract of Chilean propolis, isolated polyphenols	Clinically isolated *S. mutans* strains	In vitro-well microdilution assay, evaluation biofilm formation with fluorescence microscopy	Polyphenol mixture exhibited antibacterial activity comparable to chlorhexidine. Apigenin and pinocembrin had the lowest MIC values against *S. mutans* out of the isolated polyphenols. All samples inhibited biofilm formation, with apigenin and pinocembrin disrupting the biofilm structural integrity.	[168]
Ethanol extract of Brazilian green propolis	*P. gingivalis* ATCC 33277, W83, W50 and YH522, *P. nigrescens* ATCC 33563, *F. nucleatum* 20, ATCC 23726, *A. actinomycetemcomitans* (serotype b) Y4, ATCC 29522, *P. loescheii* ATCC 15930, *Streptococcus spp*. ATCC 33397, 51100, 10558, 6245, UA159, 9759, 10556, 6715, *E. coli* BW25113, *S. oralis* No. 10	In vitro-well microdilution assay, biofilm formation assay, membrane permeability analysis,	Extract exhibited stronger antibacterial effect against *P. gingivalis*, than against other oral bacteria. Extracts had a rapid bactericidal effect caused by disruption of cell membrane and bleb formation. The active compounds were determined as artepillin C, baccharin, and ursolic acid. Formation of biofilm was inhibited at sub-MIC concentrations.	[186]
Magnetite nanoparticles functionalized with ethanol extract of Moroccan propolis in combination with chloramphenicol	Methicillin sensitive *S. aureus* ATC 6538, clinical isolates of methicillin resistant *S. aureus*	In vitro-well microdilution assay	Functionalized magnetite nanoparticles exhibited strong antibacterial effect against both methicillin sensitive and resistant *S. aureus*. Nanoparticles with both the propolis extract and chloramphenicol exhibited complete inhibition of bacterial growth after 2 h in 2 MRSA strains. The mechanism of action was determined to be the disruption of cell wall structure and cytoplasm leakage.	[187]
Ethanol extract of Italian propolis in combinations with antibiotics	Clinically isolated strains: *S. aureus*, *S. epidermidis*, *S. hominis* strains, *S. haemolyticus*, *S. warnerii*, *S. capitis*, *S. auricularis*, *S. faecalis*, *S. viridans*, *S. β-haemolyticus*, *S. pneumoniae*	In vitro-agar dilution assay, lipase test, coagulase test, propidium iodide uptake test, adherence test	Extract exhibited strong antimicrobial activity, caused by membrane disruption. It inhibited virulence factors, reducing lipase activity and completely suppressing coagulase activity in *Staphylococcus* spp. All tested antibiotics apart from erythromycin and ceftriaxone exhibited synergistic effect with the propolis extract, especially ampicillin, gentamicin and streptomycin MIC_90_ values were reduced up to 250 times.	[188]
Ethanol extracts of Polish propolis	clinically isolated coagulase positive *S. aureus* strains and reference *S. aureus* strains, methicillin sensitive ATCC 25923 and methicillin resistant ATCC 43300	In vitro-disk diffusion assay, broth microdilution assay	Polish propolis exhibited antibacterial activity against both MSSA and MRSA. Significant synergistic effects were observed in combinations with cefoxitin, clindamycin, tetracycline, tobramycin, linezolid, trimethoprim/sulfamethoxazole, penicillin and erythromycin, while no synergism was found with ciprofloxacin and chloramphenicol.	[178]
Ethanol extract of commercial Brazilian propolis and a commercial antimicrobial containing gentamicin and amoxicillin	*Staphylococcus* strains isolated from Brazilian cattle	In vitro-broth microdilution assay	Extract at ½ MBC exhibited strong synergistic effect with the antibiotics, lowering the MIC and MBC values against *Staphylococcus* spp. of both gentamicin and amoxicillin by a factor of 10.	[189]
Ethanol extracts of Brazilian and Bulgarian propolis	*S. typhi* standard serovar 00238	In vitro-agar dilution assay	Both extracts showed significant antibacterial activity, the Brazilian sample showed a bacteriostatic activity, while the Bulgarian sample exhibited a bactericidal one. No synergy between the propolis samples and tested antibiotics was found.	[190]
Ethanol extract of Australian propolis	Methicillin resistant *S. aureus* ATCC 43300	In vitro-disc diffusion assay, resazurin microdilution assay, nucleic acid leakage assay, propidium iodide staining assay, resistance reversal assessment	Extract exhibited activity against methicillin resistant *S. aureus*, disrupting cell wall and membrane. At ½ MIC and 1MIC concentrations respectively, the extract significantly reduced the expression of PBP2a and activity of β-lactamase, inhibiting the main mechanisms of antibiotic resistance found in MRSA. At ½ MIC formation of the bacterial biofilm was inhibited.	[176]
Ethanol extract of propolis (Sigma P8904)	Methicillin resistant *S. aureus* ATCC 33591	In vitro-broth microdilution assay In vivo-rabbit nasal colonization model, examination of polymorphonuclear leukocyte count	Both the propolis extract drops and topical mupirocin treatment significantly inhibited colonization at MIC concentration. Group receiving both treatments produced the least bacteria from nasal cultures, as well as the lowest PMNL count.	[191]
Ethanol extracts of German, Irish and Czech propolis, Aqueous extract of German propolis	Reference strains: Gram-positive, gram-negative, *Candida* spp. Clinically isolated: methicillin resistant *S. aureus* strain, *K. pneumoniae* strains and *Candida* spp. strains	In vitro-broth microdilution assay, checkerboard dilution assay, time-kill assay,	All evaluated extracts exhibited significant antibacterial activity against Gram-positive bacteria, including methicillin and vancomycin resistant strains. Against Gram-negative bacteria, the ethanol extracts were shown to be moderately effective, except for *P. aeruginosa* which proved resistant. Against *Candida* spp. Irish and Czech samples exhibited a fungicidal effect, while German samples were fungistatic. Irish propolis exhibited strong synergistic activity with vancomycin, oxacillin and levofloxacin against *S*. *pyogenes*, MRSA and vancomycin resistant Enterococcus.	[175]
Ethanol extract of Chinese propolis	Methicillin resistant *S. aureus* ATCC 43300	In vitro-broth microdilution assay, checkerboard assay, nucleic acid leakage assay, live/dead staining assay, β-lactamase activity test	The combinations of the propolis extract with ampicillin and oxacillin exhibited strong synergistic effect in antibacterial activity against MRSA. Resistance reversal analysis showed that at ¼ MIC the extract reduced expression of PBP2a and the β-lactamase activity. Extract also caused cell wall and membrane damage.	[192]
Korean propolis in composite nanoemulsion with PVA and chitosan	Methicillin resistant *S. aureus* ATCC 33591, C. perfingens NCTC 8237	In vitro-broth microdilution assay, assessment of biofilm formation In vivo-rat wound infection model	Composite exhibited antibacterial properties against both strains comparable to azithromycin in vitro. At high concentrations of propolis, the composite effectively inhibited biofilm formation, causing its complete destruction. The in vivo study showed the propolis composite to have an ameliorative effect, accelerating wound curing and decreasing MRSA infection.	[193]
Ethanol extracts of Australian propolis	*S. aureus* ATCC 25923, *K. pneumoniae* ATCC 13883, *C. albicans* ATCC 10231	In vitro-agar diffusion assay, broth microdilution assay	Extracts exhibited bactericidal activity against *S. aureus*, no activity against Gram-negative or yeast strains was detected. The effect against staphylococci was determined to be the result of C-geranyl flavonoids and triterpenoids in the propolis	[165]
Ethanol extracts of Greek and Cypriot propolis	*S. dysenteriae* NCTC 2966, *S. typhimurium* NCTC 12023, *E. aerogenes* NCTC 10006, *Y. enterocolitica* NCTC 10460, *E. coli* NCTC 09001, *S. aureus* NCTC 6571, ATCC 25923 *S. epidermidis* NCTC 11047, B. cereus NCTC 7464, ATCC 9139, *L. monocytogenes* NCTC 10357, ATCC 7644, *C. tropicalis* ATCC 13801 *C. albicans* ATCC 10231, *L. bulgaricus* ACA-DC 101 L. fermentum F 12, L. casei LC 14, L. delbrueckii LDD-C1, L. plantarum LP 101, La. helveticus LH 09	In vitro-agar diffusion assay	Extracts exhibited a broader spectrum of antimicrobial activity than nisin. Tested samples had the strongest antibacterial properties against Gram-positive strains. *Lactobacillus* spp. strains were resistant to the activity, indicating selectivity beneficial for probiotic preservation.	[194]
Ethanol extracts of Anatolian propolis	*S. mutans* ATCC 25175, *S. aureus* 6538-P, S. sobrinus ATCC 33478, *S. epidermidis* ATCC 12228, *E. faecalis* ATCC 29212, *M. luteus* ATCC 9341. *P. aeruginosa* ATCC 27853, *E. coli* ATCC 11230, *S. typhimurium* CCM 5445, *E. aerogenes* ATCC 13048, *C. albicans* ATCC 10231, *C. tropicalis* ATCC 665 and *C. krusei* ATCC 6258	In vitro-broth macrodilutions assay	All extracts exhibited a potent antibacterial effect against Gram-positive bacteria. Less activity was achieved against Gram-negative strains, especially *P. aeruginosa* and *S. typhimurium*. The sample from Bursa proved the most effective, strongly inhibiting *Candida* spp. and oral pathogens, suggesting clinical potential in dental care. Total flavonoid content was shown to be correlated with antimicrobial potency of propolis	[195]
Ethanol extracts of Serbian propolis	*S. epidermidis* ATCC 14990, *S. aureus* ATCC 25923, S. sciuri ATCC 29062, *E. faecalis* ATCC 29212, B. subtilis, *L. monocytogenes* SLCC 2375. *E. coli* ATCC 25922, *P. aeruginosa* ATCC 27853. *S. marscenscens, P. stuartii, C. guilliermondii, C. parapsilosis, C. albicans*	In vitro-agar diffusion assay, agar dilution assay, synergy disc diffusion assay	Extracts exhibited strong antimicrobial activity against Gram-positive bacteria and fungi, while Gram-negative species were not inhibited. Antimicrobial effect of propolis was not affected by antibiotic resistance. At subinhibitory concentrations extracts exhibited strong synergism with ceftriaxone against *K. pneumoniae* and nystatin against *C. albicans*.	[196]
Ethanol extracts of Brazilian and Bulgarian green propolis	*S. typhi* standard serovar 00238	In vitro-agar dilution assay, synergism assay	Extracts of Brazilian propolis exhibited bacteriostatic activity while extracts of Bulgarian propolis were bactericidal to *S. typhi*. Synergism study showed significant increase in antibacterial effect of β-lactam antibiotics when combined with either propolis sample at sub-MIC concentrations	[197]
Ethanol extracts of green propolis	*C. albicans* ATCC 443-805-2, *C. parapsilosis* ATCC 726-42-6, *C. tropicalis* ATCC 1036-09-2	In vitro-disc diffusion assay, biofilm formation assay	Extract exhibited dose-dependent growth inhibition of all tested *Candida* spp. Biofilm formation was significantly inhibited at low concentration of the extract	[198]

### 3.4. Antimicrobial Activity of Bee Venom

In recent years, bee venom has gathered scientific interest for its potent antimicrobial effects against bacteria and fungi [19] (Table 3). While compositions of bee venom from different sources vary, it has been shown that this activity is largely caused by the activity of peptides and enzymes present in the substance [199].

One of the best researched of them, melittin, a small 26-amino-acid cationic peptide that constitutes around half of apitoxins dry weight [200]. It is α-helical and amphipathic in character, which allows it to bind and insert into the lipid bilayer, creating pores and allowing leakage of cytosolic content and ions [201]. Such loss of membrane integrity leads to rapid lysis of the cell. The effectiveness of this mechanism against bacteria has been shown to be dependent on the cell envelope structure—due to lack of outer membrane, Gram-positive bacteria exhibit greater sensitivity to the cytolytic activity of melittin than Gram-positive bacteria [47], which while still susceptible to effect, often require significantly higher concentrations to be affected [47]. Similar to bacteria, fungal cell membranes can be disrupted by melittin. Fungal membranes contain ergosterol and a higher proportion of negatively charged lipids compared to mammalian membranes, making them good targets for cationic peptides [202]. Melittin has been shown to directly permeabilize yeast cell membranes, causing leakage of vital contents. For instance, melittin exhibits fungicidal activity against *Candida albicans* by causing the cells to rapidly lose membrane integrity and viability [203]. Whole BV is often even more potent: one study reported BV completely inhibited growth of Trichophyton mentagrophytes within 5 min at 15–30 µg/mL, while the standard antifungal drug fluconazole was ineffective [204]. A very promising aspect of BV components is their synergistic interaction with existing antibiotics. In synergy assays, melittin drastically lowers the MIC of various antibiotics against resistant bacteria. For example, melittin combined with oxacillin was bactericidal against MRSA that oxacillin alone was not able to affect [205]. Another study found that a combination of melittin and doripenem reduced the required concentrations of each > 60-fold against MDR A. baumannii. Similar synergy was seen for melittin with colistin and with ceftazidime against *P. aeruginosa* [206]. Beyond membrane damage, melittin can trigger apoptotic-like cell death in fungi. Researchers have observed that *Candida albicans* cells exposed to sub-lytic doses of melittin exhibit symptoms of apoptosis: DNA fragmentation, phosphatidylserine externalization on the cell membrane, and activation of caspase-like proteases [207]. Melittin treatment led to a surge in intracellular ROS, which in turn caused mitochondrial dysfunction in *C. albicans*. Specifically, melittin disrupts the fungal mitochondrial membrane, leading to release of calcium ions and activation of a caspase-dependent death pathway [208].

Phospholipase A_2_: PLA_2_ on its own has relatively modest antibacterial effects compared to melittin –reported minimum inhibitory concentrations of PLA_2_ are typicallu much higher than those of melittin [209]. However, PLA_2_’s activity dramatically increases in the presence of melittin. Melittin-induced membrane pores and deformations expose the inner phospholipids of bacterial membranes, providing access for PLA_2_ to its substrate [210]. This cooperative action results in complete membrane disintegration. PLA_2_ likely contributes to antifungal action as well by digesting fungal membrane phospholipids [211]. Fungi have an outer cell wall made of glucans and chitin and melittin can penetrate this cell wall, after which PLA_2_ can reach the membrane. The enzymatic breakdown of membrane lipids by PLA_2_, together with melittin’s pore formation, leads to cell lysis [210]. Other minor peptides in BV, such as secapin, may have auxiliary antibacterial effects or could potentiate the action of melittin. MCD peptide, for instance, causes release of histamine from host mast cells, which might indirectly create an inflammatory environment unfavorable for pathogens [212]. Some BV peptides also exhibit protease inhibition or other activities that could stress bacteria. However, detailed mechanisms for these lesser components are less documented, and their roles are presumably supportive [207].

**Table 3:** Selection of articles on the antimicrobial effect of bee venom.

Material	Microorganism	Assay	Key Results	Reference
Commercial bee venom samples	*E. coli* k-12 ATCC 47074, *P. putida* ATCC 7000008, *P. fluorescens* NCIMB 9046	In vitro-bacterial viability assay	Venom samples exhibited a strong inhibitory effect on *E. coli*, with viability decreasing proportionally to the increase in venom concentration. Antibacterial activity against *P. putida*, while present, did not increase with concentration beyond 225 µg/mL. No effect against *P*. *fluorescens* was observed. Cell membrane damage and pore formation were determined as the mechanism of action.	[213]
Commercial bee venom and in natura samples, melittin and phospholipase A2	*S. salivarius* ATCC 25975, *S. sobrinus* ATCC 33478, *S. mutans* ATCC 25175, *S. mitis* ATCC 49452, *S. sanguinis* ATCC 10556, *L. casei* ATCC 11578, *E. faecalis* ATCC 4082	In vitro-broth microdilution assay	Both commercial and in natura apitoxins exhibited strong antimicrobial effects. Phospholipase A2 did not inhibit growth of tested strains, except for L. casei, which was inhibited at high concentrations. Melittin exhibited the highest level of activity against all tested strains.	[209]
Bee venom and melittin samples from *A*. *dorsata*, *A*. *mellifera*, *A*. *florea*, and *A*. *cerana* species	*S. aureus* TISTR 517, *S. epidermidis* DMST 15505, methicillin-resistant *S. aureus* DMST 20625, *B. subtilis* DMST 15896, *M. luteus* DMST 15503, *K. pneumoniae* DMST 8216, *S. typhimurium* DMST 562, and *E. coli* ATCC 25922, *C. albicans* TISTR 5554	In vitro-broth microdilution assay	All tested venom and melittin samples exhibited low to none antimicrobial activity against Gram-negative bacteria strain. The inhibitory effect against Gram-positive bacteria, while present against all strains, did not show significant differences between the venom samples and their respective melittin activity, except for A. dorsata venom which inhibited MRSA growth stronger than melittin. *A*. *mellifera* and *A*. *cerana* venoms inhibited *C. albicans* growth, despite yeast proving resistant to all tested melittins.	[47]
Collected bee venom	Clinical isolates of *S. agalactiae*, *S. gordonii*, *S. epidermidis*, *S*. *bovis S. aureus*, methicillin resistant *S. aureus. S. pneumonia* laboratory strain.	In vitro-broth microdilution assay In vivo-mouse infection model	Bee venom exhibited strong antibacterial activity against all tested strains. While active against MRSA strains, in vivo administration of bee venom enhanced MRSA propagation. Melittin exhibited a superior effect on survivability of MRSA infected mouse compared to bee venom.	[214]
Bee venom and isolated melittin	*S. aureus* ATCC 13464, ATCC 14558, ATCC 19095, ATCC 23235, methicillin resistant *S. aureus* clinical isolates	In vitro-resazurin microdilution assay.	Both venom and melittin exhibited similar potent antibacterial effect against tested *S. aureus* strains. Neither apitoxin nor melittin affected bacterial enterotoxin production. Both apitoxin and melittin enhanced the activity of oxacillin. Exposure of MRSA strains to apitoxin and melittin caused extensive morphological changes to the bacteria.	[205]
Synthetic melittin	Clinical isolates: *S. aureus* and *P. aeruginosa*. *S. aureus* ATCC 25923, ATCC 29213, P aeruginosa PAO1	In vitro-broth microdilution assay, biofilm formation test, synergy assay	It was show melittin, alone and in combination with conventional antibiotics has a strong antibacterial effect against tested MDR pathogens as well as their mature biofilms. Synergistic effect with antibiotics at low concentrations was demonstrated.	[206]
Bee venom from 5 apiaries in Equador	*S. enterica* and *L. monocytogenes* strains, including S. enterica CECT 4395 and *L. monocytogenes* CECT 934	In vitro-broth microdilution assay	All apitoxins exhibited similar antibacterial effects against *Salmonella* spp. strains. Inhibitory activity was significantly stronger against *L. monocytogenes* strains.	[215]
Bee venom extracts in DMSO	isolates from wastewater near hospitals—*P. mendicina, K. pneumonia* and *E. coli* MDR strains	In vitro-disc diffusion assay, agar dilution assay	Apitoxin exhibited significant antimicrobial activity against Gram-negative bacteria. All tested antibiotics had increased effectiveness when combined with bee venom, independently of the strain of bacteria.	[216]
Bee venom loaded on chitosan nanoparticles	clinically isolated strains: *K. ohmeri*, *C. neoformans* and *C. albicans* ATCC90023 reference strain	In vitro-agar well diffusion assay, yeast-hypheal transition study	The bee venom loaded nanoparticles exhibited significantly higher levels of antifungal activity against *C*. *neoformans* and *C. albicans* than free nanoparticles. The nanoparticles effectively inhibited the formation of biofilm of all isolates. Disruption of yeast-hypheal transition was determined in all isolates.	[217]
Bee venom loaded on chitosan nanoparticles	*E. coli* ATCC 8739, *P. aeruginosa* ATCC 9027, *B. subtilis* ATCC 6633, *S. aureus* ATCC 7984	In vitro-agar well diffusion assay, broth macrodilution assay	The bee venom loaded nanoparticles exhibited an inhibitory stronger than that of either free nanoparticles or bee venom against all tested strains. Bactericidal effect was improved by bee venom loading only against *S. aureus*.	[218]
Collected bee venom	Methicillin resistant *S. aureus* CCARM 3366, CCARM 3708	In vitro-broth microdilution assay, checkerboard assay	Bee venom exhibited a strong antibacterial effect against tested MRSA strains. Significant synergistic effects have been determined in combinations of bee venom with gentamycin ant vancomycin.	[219]

## 4. Antiviral Activity of Bee Products

### 4.1. Viral Infections

These natural substances, produced by honeybees, have been used in traditional medicine for centuries and are now being investigated in modern research as potential antiviral agents [19,220]. A wide range of human pathogenic viruses have been found to be susceptible to varying degrees to components of bee products. Reported targets include respiratory viruses, herpesviruses, human immunodeficiency virus (HIV), hepatitis viruses, and enteroviruses, among others. Bee products can exert antiviral effects through multiple mechanisms: direct virucidal action, inhibition of virus entry into host cells, suppression of viral replication inside cells, and modulation of the host immune response to infection [220].

The global disease burden of viral infections is immense and variable by pathogen and region. HIV/AIDS remains a leading viral pandemic: WHO estimates ~39.9 million people were living with HIV at the end of 2023, with roughly 1.3 million new infections and 630,000 HIV-related deaths occurring in that year [221,222]. Over the course of the HIV pandemic, some 88.4 million people have been infected. The successful scale-up of antiretroviral therapy since the 2000s has reduced HIV mortality by ~50% since 2010, but the number of people living with HIV continues to grow as treatments prolong survival [221,222]. Influenza causes recurring seasonal epidemics worldwide. WHO reports that seasonal influenza infects roughly one billion people globally each year, including 3–5 million severe cases. Annually it leads to 290,000–650,000 respiratory deaths, predominantly in the very young, elderly, or those with comorbidities [223]. Over 99% of influenza-attributed child deaths occur in low- and middle-income countries, reflecting inequities in healthcare [224]. Pandemic influenza can add additional global morbidity and mortality beyond seasonal levels, although no novel pandemic strain has emerged since 2009 [225]. The SARS-CoV-2 pandemic of 2020–2022 produced by far the largest global outbreak of a new viral disease in recent history. As of 2024, WHO reported over 776 million confirmed COVID-19 cases and more than 7 million deaths globally [226]. The vast majority of cases have occurred in the Americas, Europe and Asia, with fewer reported cases in Africa but the true number of infections is known to exceed confirmed case counts due to under-reporting and asymptomatic cases [227,228]. After peaking in 2020–2021, annual SARS-CoV-2 mortality has declined, but resurgence is periodic with the emergence of new variants. Chronic viral hepatitis is another major global burden [227,228]. WHO estimates that in 2022 there were ~254 million people with chronic hepatitis B (HBV) infection and ~50 million with chronic hepatitis C (HCV) [226]. During that year, ~1.2 million people newly acquired HBV and ~1.0 million acquired HCV. Chronic HBV and HCV together cause roughly 1.3 million deaths per year from cirrhosis and liver cancer [226].

### 4.2. Antiviral Activity of Honey

Honey’s specific chemical constituents of honey have demonstrated direct antiviral effects and immune-modulatory actions (Table 4). Studies have shown that honey can inactivate enveloped viruses directly. For example, natural honeys have proven virucidal against several enveloped viruses, including herpes simplex virus (HSV), varicella zoster virus (VZV), influenza viruses, and HIV. In one notable in vitro study, Manuka honey exhibited potent activity against influenza A virus, with no cytotoxic effects at effective doses [229], while exhibiting strong synergystic properties in combinations with oseltamivit and zanamavir. This antiviral effect was attributed to direct virucidal action on the virus and was strong enough that combining a sub-therapeutic concentration of Manuka honey with the antiviral drugs oseltamivir or zanamivir reduced the drugs’ necessary concentration 1000-fold, indicating a strong synergistic effect. Honey has also shown activity against HIV-1 in cell culture: a panel of eight monofloral Iranian honeys caused significant dose-dependent suppression of HIV replication in infected human peripheral blood mononuclear cells [230]. Analysis identified methylglyoxal as a key anti-HIV component; methylglyoxal extracted from these honeys inhibited HIV at a late stage of the viral life cycle. There is evidence of honey inhibiting HSV-1 and HSV-2 and VZV [229]. Honey’s sugars and hydrogen peroxide provide broad antimicrobial action, while specialized components like methylglyoxal, flavonoids, and defensin-1 target viruses more specifically. These compounds can bind viral proteins or genomes and block the processes of infection. For instance, flavonoids in honey such as quercetin have been shown to bind viral enzymes; quercetin can inhibit viral proteases [156].

### 4.3. Antiviral Activity of Propolis

In the context of viruses, propolis has demonstrated remarkably broad antiviral effects across diverse virus families (Table 5). Extensive in vitro studies have confirmed that propolis extracts can inhibit many human viruses. For example, propolis preparations have shown potent activity against herpesviruses [242,243]. In one study, propolis flavonoids not only inhibited HSV replication on their own but also showed synergy when used with the drug acyclovir [242]. The combination of propolis extract with acyclovir was more effective at suppressing HSV-1/2 than acyclovir alone, hinting that propolis components may attack additional viral or host targets to complement the drug’s action. Propolis is also effective against VZV [244]. Studies have shown that propolis can inhibit influenza viruses [245]. Some early research indicated propolis flavonoids could reduce the infectivity of adenovirus, and a propolis extract showed moderate inhibition of rhinovirus replication [246]. Clinical trials showed propolis to have a significant positive effect on the recovery of COVID-19 patients [247]. Though not a standalone cure, propolis helped reduce lung damage markers and viral load, supporting its role as an immune-supportive antiviral adjunct. Flavonoids and phenolic acids in propolis can block viral adsorption and entry by attaching to viral surface proteins or host cell receptors. They can also inhibit viral enzymes: for example, apigenin, kaempferol and quercetin from propolis have been shown to inhibit the DNA polymerase of herpesviruses and the RNA polymerase of influenza virus [248]. Moronic acid, a triterpenoid from Brazilian propolis, exhibited sub-micromolar potency against HIV-1 in cell culture by inhibiting HIV protease activity [249]. Propolis is also a known immunomodulator that can enhance the host’s antiviral response. In HIV-infected cell models, propolis not only suppressed viral replication but also modulated cytokine secretion to a profile more effective at combating the virus [250].

### 4.4. Antiviral Activity of Bee Venom

It has been demonstrated that non-cytotoxic concentrations of whole bee venom or isolated melittin can significantly inhibit the replication of multiple viruses (Table 6). It has been reported that honey bee venom and melittin, when co-incubated with virus, blocks the infectivity of influenza A virus, vesicular stomatitis virus (VSV), RSV, and HSV in vitro [262] and in vivo, melittin loaded nanoparticle administration protects mice from an influenza A (H1N1) infection, by piercing the viral envelope and subsequently destroying the virus. Bee venom has also shown activity against hepatitis C virus (HCV) even at low concentrations exhibiting a potent virucidal effects on HCV [263]. Similarly, melittin and PLA_2_ from venom have been reported to inactivate other viruses like HIV and porcine pseudorabies virus [264]. It is important to note that while melittin is the most powerful antiviral component of apitoxin, whole bee venom’s other peptides which might have ancillary antiviral effects or help melittin’s action. In one comparative study, a fraction of venom containing PLA_2_ has been shown to reduce influenza virus infectivity as well, though less than melittin [265]. Beyond direct virucidal action, bee venom components can modulate the immune system stimulating the production of anti-inflammatory cytokines and promoting regulatory T-cells [266].

## 5. Wound Treatment—A Promising Use of Bee Products in Medicine

Wounds resulting from burns, injuries, incisions, or medical procedures involve disruption of the skin continuity and disruption of the body’s natural barrier exposes tissues to colonization by various microorganisms, increasing the risk of infection [271]. Physiological wound healing aims to restore tissue integrity, but effective regeneration of the skin tissue remains a serious problem, as complications that occur during this process can lead to health consequences. The wound healing process in the body consists of many biochemical and cellular reactions and is divided into several stages: hemostasis, inflammation, proliferation, and remodeling [272]. Hemostasis is defined as the immediate response to injury and involves vasoconstriction and clot formation to limit blood loss. In the inflammatory phase occurs the infiltration of neutrophils and macrophages which eliminate cellular debris and pathogens. The initiation of tissue regeneration begins with the proliferation phase through fibroblast activity, angiogenesis and keratinocyte migration. The final remodeling phase involves the reorganization of collagen fibers and wound scarring [272,273].

The oldest report documenting the use of bee products as a medical agent came from Egypt 4000 years ago [274]. Modern studies provide increasing evidence to support the efficacy of bee products in promoting and accelerating wound healing [275]. For example, Takzaree et al. [276] showed that local application of thyme honey affected key processes such as shortening the inflammatory phase, stimulating the formation of granulation tissue, promoting angiogenesis, early onset of cell proliferation, remodeling, and ultimately accelerating the healing of open wounds in rats [276]. The therapeutic properties of honey result not only from its widely described antimicrobial effects but also from its physicochemical characteristics [276]. One of the most meaningful properties of honey is its influence on maintaining a moist wound environment. This wet environment prevents scab formation, alleviates necrotic changes in the dermis, and increases the migration of keratinocytes to the lesion surface, accelerating re-epithelialization [277,278]. In addition, the high sugar content contributes to the osmotic effect, which inhibits bacterial growth and attracts water and lymph to the wound area, contributing to better nutrition of the damaged tissue and strengthening the immune response. In addition, the low pH of honey provides an acidic microenvironment that promotes fibroblast activity and epithelial regeneration. In turn, the high viscosity of honey provides a protective barrier against external contamination, reducing the risk of microbial penetration [279]. Interestingly, in addition to many reports on the ability of honey in supporting superficial wound care, its effectiveness as a skin graft fixative in patients with burns has also been described. It has been shown [280] that using of honey not only reduced the frequency of infections and pain reported by patients but also improved the adhesion of the split-thickness graft and overall healing results.

The properties of propolis make it a promising agent for the treatment of wounds of various origins like infected wounds, acute injuries, but also thermal wounds (burns and frostbite), as summarized Yang et al. [281]. The therapeutic effect of propolis is not only related to its antimicrobial and anti-inflammatory activity but also its analgesic effect conditioned by the presence of the flavonoid—chrysin [31,274]. Moreover, propolis has been shown to promote the proliferation and migration of keratinocytes and fibroblasts, which supports the regeneration of the epidermis and dermis, while reducing excessive scar formation [282]. Studies have shown that propolis has an effect at various stages of the healing process—from the inflammatory to tissue remodeling. Propolis has been shown to inhibit the expression of pro-inflammatory cytokines such as TNF-α, IL-1β, or IL-6 [283], but also to increase the level of anti-inflammatory cytokines [283] and growth factors such as VEGF [284], which promotes the formation of new tissue. Importantly, propolis does not have a toxic effect on skin cells and rare allergic reactions, which makes it a safe therapeutic agent for topical use [33]. The clinical use of propolis has been confirmed, among others, in the treatment of diabetic foot ulcers [285]. A randomized controlled trial showed that local application of propolis significantly shortened the healing time of wounds and reduced the degree of infection in patients with type II diabetes [286]. Similar effects in the treatment of diabetic wounds were also obtained in the study of the effect of bee venom. Hozzein et al. [287] reported that bee venom treatment improved wound closure in mice with type I diabetes, restored antioxidant enzyme activity, normalized chemokine levels, and protected wound macrophages from apoptosis. This indicates the immunomodulatory and cytoprotective effects of bee venom under oxidative stress, which is typical for chronic wounds in patients with diabetes [288].

Positive results have also been demonstrated with bee pollen. Olczyk et al. [289] reported that using bee pollen ointment had a positive effect on the healing of burn wounds while improving the general condition of the animals in a pig model, and showed strong antimicrobial properties, preventing secondary infections. The therapeutic effects of royal jelly have been well described in the context of many diseases, including diabetic foot ulcers [290]. Although the effect of this product on wound healing is not fully understood, available data suggest significant pro-regenerative properties. Kim et al. [291] conducted a study in which they assessed the effect of royal jelly on human fibroblasts in vitro because the migration of these cells is one of the initial phenomena during the formation of new skin tissue. The results showed that royal jelly increased fibroblast migration and influenced the expression of signaling lipids involved in the wound healing process, indicating a potential role in initiating skin repair [291]. Promising results were obtained by Alvarez et al. [292] in a study in which the effect of royal jelly extracellular vesicles (RJEV) on wound healing was assessed in a mouse model. As a result, RJEV was shown to have antibacterial activity and significantly accelerated the initial wound closure process [292].

The current state of knowledge indicates that combining different bee products produces a synergistic effect in the wound healing process. Andritoiu et al. [293] conducted a study evaluating the efficacy of an ointment based on honey, propolis, drone brood homogenate and a mixture of these substances on different types of wounds: incisions, excisions and burn wounds, in an animal model. The results showed that all the products used accelerated wound shrinkage and re-epithelialization, and the best results were observed after using an ointment containing a combination of all apitherapeutics. In addition, it was shown that combining bee products with other natural agents also has beneficial effects. For example, Javadi et al. [294] showed that the best therapeutic properties on wound healing in rats were obtained after using a mixture of honey and Nigella sativa seed oil compared to using each of these substances separately. Also, a study by Bayir et al. [295] showed that the use of a dressing impregnated with beeswax, olive oil, and butter in a rat model of second-degree burns improved the regeneration of the dermis and epidermis, increased fibroblast activation and keratinization, and had a positive effect on wound contraction [295]. The above data indicates that using the synergistic effect of bee products and other natural substances has therapeutic potential as a comprehensive approach to wound treatment.

## 6. Obstacles on the Way to Implementing Bee Product Treatments in the Current Medical Landscape

Medicinal use of honey is generally considered safe [19]. It has been shown although it has been shown that about 5% of patients treated with topical medical honey report pain at the application site, more than with conventional dressings [296]. On the other hand, serious adverse effects from honey are rare, there are no severe systemic reactions to medical-grade honey reported in the literature. Propolis is a well-known contact sensitizer, and allergic reactions are a significant concern. Repeated topical or oral exposure can lead to sensitization in susceptible individuals. Allergic manifestations include contact cheilitis, oral mucositis, perioral eczema, labial edema, and even dyspnea in severe cases. Patch test studies have shown that 1–6% of adults tested exhibit sensitivity to propolis; higher rates have been reported in certain populations like children with eczema [297]. Systemic effects such as acute renal failure [298] associated with high dose propolis ingestion have been reported. Bee venom therapy carries the most serious safety risks. Bee venom is a complex mixture of peptides and enzymes that can trigger profound immunological reactions. Allergic and anaphylactic reactions are the paramount concerns. A comprehensive 2015 systematic review of 145 studies found that systemic adverse reactions occurred in about 14% of patients undergoing bee venom therapy, including numerous cases of anaphylaxis, with some requiring emergency epinephrine and steroids. There has been at least one documented fatality directly attributed to bee venom apitherapy [299]. Apart from anaphylaxis, other severe adverse events recorded include hemolysis and even a case of Guillain–Barré syndrome and an irreversible nerve injury [300]. Repeated exposure also carries the risk of sensitization: someone may develop an allergy after multiple bee venom treatments [299]. Overall, the clinical risk-benefit profile of bee venom is problematic, the unpredictable severity of reactions has thus far limited its acceptance in mainstream infection management. In the context of treating infections, where safer alternatives exist, the threshold for tolerating venom’s risks is especially high.

Traditionally venom delivered by the bee’s own mechanism or by subcutaneous injections of collected venom [301]. Using live bee stings is not precise in dosage, as each bee may inject a slightly different amount of venom and the process cannot be standardized clinically [302]. Injections with purified venom allow precise dosing, but venom proteins are prone to degradation, so they are often lyophilized and reconstituted fresh for use [303]. Another pharmacologic issue is maintaining potency during storage. Honey can lose enzymatic activity if improperly stored [304], propolis extracts can undergo chemical changes that not only reduce efficacy but may increase allergenicity [297] and dried bee venom can lose some volatile components over time [305]. Unlike single-compound drugs, bee products have multiple active constituents that work in concert [19] and their metabolites could be different from the parent compounds that showed activity in vitro.

Bee products can inadvertently contain harmful contaminants from the environment. Honey and propolis can accumulate pesticide residues and heavy metals from flowers and sap if hives are near polluted areas. Studies have shown that propolis can serve as a bioindicator of environmental pollution. Propolis from industrial or mining regions often contains elevated concentrations of lead, cadmium or arsenic [306]. Propolis and pollen products, if not properly processed, might carry fungal spores or bacteria from the hive. To be medicinal grade, these products must be processed in sterile conditions or sterilized without degrading active ingredients [307].

## 7. Conclusions

**Figure 1:**
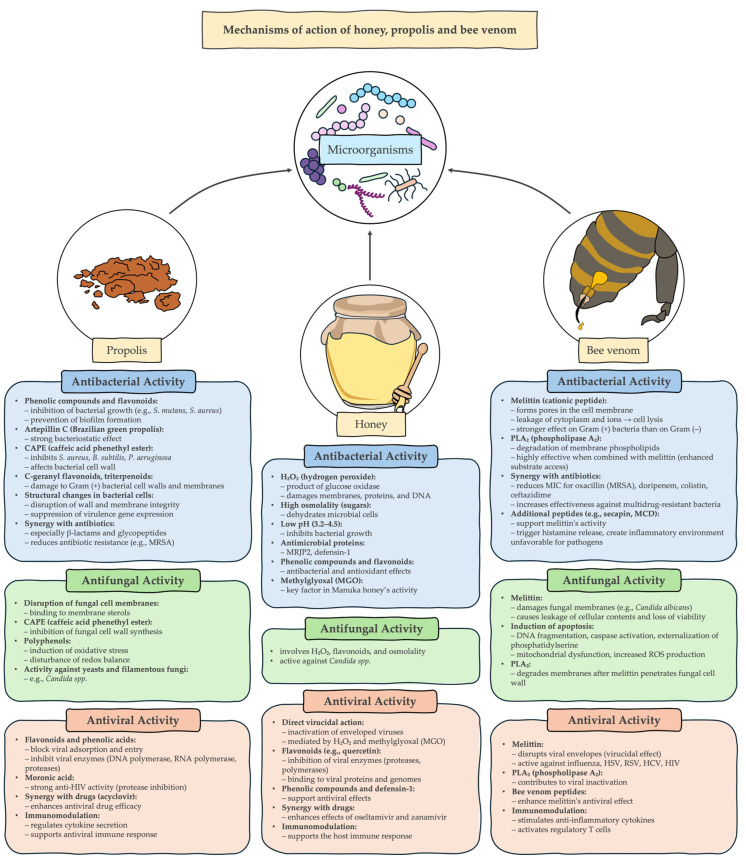
Mechanisms of action of honey, propolis and bee venom.

This review highlights the significant potential of bee products to support and complement modern therapies for the treatment of infectious diseases. Honey, propolis and bee venom show a broad spectrum of antimicrobial activity (Figure 1), including efficacy against multi-drug resistant (MDR) microorganisms, making them a promising target for further research. Their synergy with conventional antibiotics and their documented use in wound healing strengthen the position of apitherapeutics in regenerative and infectious medicine. Despite the existing regulatory and technological barriers, the integration of bee preparations into modern therapeutic strategies can provide significant health benefits. Further clinical trials and the development of standardized formulations are needed to fully exploit the therapeutic potential of these natural substances.

## 8. Methodology of the Literature Search

A comprehensive literature search was conducted in the PubMed database to identify scientific papers on the antimicrobial properties of honey, propolis, and bee venom. No time limits were applied to ensure a complete review of the available data. The search strategy included a combination of terms related to bee products and their biological activity, mechanisms of action, and potential use in combating infections.

The search was based on the following phrases: (honey OR propolis OR bee venom) AND (antimicrobial OR antibacterial OR antiviral OR antifungal OR anti-infective OR antimicrobial resistance OR pathogen inhibition OR biofilm inhibition) AND (mechanism of action OR biological activity OR therapeutic application OR pharmacological properties OR infection treatment OR synergistic effect).

Additionally, extended queries were used, such as: (honey OR propolis OR bee venom) AND (drug resistance OR antibiotic resistance OR multidrug-resistant pathogens OR alternative therapy OR natural antimicrobials). To enhance the completeness of the review, results from Google Scholar were also included and a hand search of journals in the fields of microbiology, pharmacology, apitherapy and natural medicine was performed.

The selection process was conducted independently by two researchers. In the case of papers with similar topics or results, priority was given to the most up-to-date ones, containing reliable data on the chemical composition, mechanisms of action and antimicrobial properties of honey, propolis and bee venom, as well as their potential use in the treatment of infections.

## Figures and Tables

**Table 1:** Selection of articles on the antimicrobial effect of honey.

Material	Microorganism	Assay	Key Results	Reference
Commercial therapeutic honeys: Manuka (*Leptospermum scoparium*), Rewa Rewa (*Knightia excelsia*), Medihoney™ Local honeys: Lavender (*Lavandula x allardii*), Red Stringybark (*Eucalyptus macrorrhyncha*), Paterson’s Curse (*Echium plantagineum*)	*C. albicans*, *A. faecalis*, *C. freundii*, *E. coli*, *E. aerogenes*, *K. pneumoniae*, *M. phlei*, *S. california*, *S. enteritidis*, *S. typhimurium*, *S. marcescens*, *S. sonnei*, *S. aureus*, *S. epidermidis*.	In vitro—agar dilution assay	Most tested honeys showed significant growth inhibition at 10–20% against all tested pathogens, except *C. albicans* and S. marcescens. Commercially therapeutic honeys outperformed local honeys. Medihoney™, manuka and red stingybark honeys were most effective against *S. aureus* and *E. aerogenes* strains.	[142]
80 Australian *Leptospermum* spp. honeys	*S. aureus* ATCC 25923	In vitro—catalase treated disc diffusion assay for non-peroxide antibacterial activity	The study revealed a strong correlation between methylglyoxal content and non-peroxide antibacterial activity against *S. aureus*. The methylglyoxal content of Australian *Leptosperum* spp. was found to be comparable to manuka honey from New Zealand.	[136]
Medical-grade honey formulation	Clinical isolates of *C. auris*, *C. albicans*, *C. glabrata*, *C. krusei*, *C. parapsilosis*	In vitro—broth microdilution assay	Medical grade honey sample exhibited dose-dependent antifungal activity against C. auris, with no significant reduction in activity against multiresistant strains. Full formulation exhibited stronger activity than the honey component alone.	[139]
Flavonoid extract of honey	*C. albicans* ATCC 10123	In vitro—hyphal transition evaluation, determination of intracellular glutathione, glutathione metabolism enzymes activity evaluation	The extract significantly inhibited the dimorphic conversion of *C. albicans*, reducing reactive oxygen species generation and inhibiting c-glutamyl transpeptidase activity.	[140]
Sidr honeys (Ziziphus spina-christi) with silver nanoparticles	*B. subtilis*, *E. coli*, *P. aeruginosa*, *C. albicans*	In vitro—agar diffusion assay	Three out of four honey samples exhibited significant synergistic effects with silver nanoparticles against all tested pathogen strains, except for *B. subtilis*.	[143]
Manuka and pasture honey	*S. aureus* clinical isolates	In vitro—agar dilution assay	All 18 coagulase-negative isolates were inhibited by both honeys at 2.7–5% (v/v) concentration, while simulated honey control, with antibacterial activity limited to the osmotic effect, inhibited growth at concentrations above 20% (v/v).	[144]
Honey from *Melipona beecheii*	*C. albicans* ATCC 1023	In vitro—agar dilution assay, broth macrodilution assay, SEM study	Honey exhibited complete inhibition of *C. albicans* growth at ≥20% (v/v) and a fungicidal effect at 35% (v/v). At sublethal concentrations the honey caused extensive morphological changes in the structure of cell wall.	[145]
*Acacia mangium* honey, *Melaleuca cajaputi* honey, Stingless bee (*Trigona* spp.) honey *Ananas comosus* honey and polyfloral *Apis dorsata* honey	*S. aureus* ATCC 25923, *B. cereus* ATCC 11778, *E. coli* ATCC 25922, *P. aeruginosa* ATCC 27853	In vitro—agar diffusion assay, non-peroxide activity assay with catalase treatment	All honeys exhibited antibacterial activity, *Melaleuca cajaputi* honey had the strongest effect against all tested strains, while *Acacia mangium* proved the least effective. Honey samples with the most potent antibacterial effect possessed high non-peroxide activity.	[146]
Manuka honey, Ulmo 90 honey (*Eucryphia cordifolia*), synthetic honey	Methicillin resistant *S. aureus* ATCC 43300 and 4 clinical isolates, *E. coli* ATCC 35218, *P. aeruginosa* ATCC 27853	In vitro—agar diffusion assay, broth microdilution assay, non-peroxide activity assay with catalase treatment	Ulmo honey exhibited superior antibacterial activity against MRSA strains, compared to manuka honey. There was no significant difference in activity against Gram-negative strains. Antibacterial activity of ulmo honey was determined to be primarily peroxide dependent.	[134]
Finnish monofloral honeys: *Epilobium angustifolium, Calluna vulgaris, Fagopyrum esculentum, Rubus chamaemorus* and *Vaccinium vitis-idaea*	S. pyogenes ATCC 8184 *S. aureus* ATCC 25923 Methicillin-resistant *S. aureus* ATCC 43300 *S. pneumoniae* (clinical isolate SB 53845)	In vitro—broth microdilution assay,	*E. angustifolium*, *C. vulgaris* and *F. esculentum* exhibited significant dose-dependent antibacterial activity, while *R. chamaemorus* and *V. vitis-idaea* demonstrated minimal to no inhibitory effect.	[133]
Manukacare 18+ manuka honey—*Leptospermum* spp.	Epidemic methicillin resistant *S. aureus* EMRSA-15 NCTC 13142, *P. aeruginosa* NCIMB 8626	In vitro-broth microdilution assay, E-strip test, disc diffusion assay and checkerboard assay for synergy assay, growth curve analysis	Honey exhibited an inhibitory effect against MRSA and *P. aeruginosa*. The synergistic effect with honey was observed for imipenem, mupirocin and tetracycline against MRSA. Against *P. aeruginosa* synergism was exhibited by tetracycline, rifampicin and colistin	[147]
37 Polish monofloral honeys	*S. aureus* PCM 2051, *S. epidermidis* PCM 2118, *P. aeruginosa* ATCC 27853, *E. coli* K12	In vitro-broth microdilution assay,	Out of the tested honey samples, cornflower, thyme, buckwheat and tansy phacelia honeys exhibited the strongest inhibitory effect, especially against staphylococci. The antibacterial effect was strongly correlated to honey color and phenolic content. Some samples presented greater antibacterial potency than manuka honey. Antibacterial activity was determined to be primarily hydrogen peroxide dependent.	[148]
16 Thai monofloral honeys	Skin disease pathogens: *S. aureus*, methicillin resistant *S. aureus*, *S. epidermidis*, *Corynebacterium* sp., *B. subtilis*, *M. luteus*, *P. acnes*, *P. aeruginosa*,	In vitro-agar diffusion assay, broth microdilution assay, time-kill assays	Out of the tested samples, longan honey exhibited the strongest antibacterial effect, especially against all tested MRSA strains. *M, luteus*, *B. subtilis*, *S. epidermidis* and *P. aeruginosa* were not inhibited by any of the honey samples.	[149]
3 Turkish monofloral honeys and one multifloral	Clinical isolates of *C. albicans*, C. glabrata, *C. krusei*, *Trichosporon* spp.	In vitro-broth microdilution assays	All analyzed samples exhibited an antifungal effect at low concentrations. According to the MIC values achieved, in terms of overall level of activity, the multifloral sample was stronger in comparison to the monofloral samples.	[150]
11 Danish floral honeys (incl. Water Mint—*Mentha aquatica*, Linden—*Tilia cordata*, Organic mix), commercial processed honey (Jakobsens), raw and medical-grade Manuka (*Leptospermum scoparium*)	*Staphylococcus aureus* (2 strains), *Staphylococcus epidermidis*, *Pseudomonas aeruginosa*, *Escherichia coli*	In vitro-agar-well diffusion assay	All honeys except commercial processed honey exhibited antibacterial activity. Water Mint, Linden, and Organic honeys showed the strongest effect, greater than Manuka honeys. Antibacterial effect correlated with hydrogen peroxide content rather than methylglyoxal.	[151]
Honey (floral origin not specified)	*Escherichia coli* ATCC 25922	In vitro-agar plate assay, liquid culture; in vivo-*E. coli*-inoculated rats	Honey inhibited *E. coli* growth in both solid and liquid media. In rats, honey feeding reduced bacterial load in feces compared to controls. Honey also increased intestinal SCFA concentration.	[152]
Honey from Iyale, Kogi State, Nigeria	*Escherichia coli*, *Pseudomonas aeruginosa*, *Streptococcus pyogenes*, *Staphylococcus aureus*, *Proteus mirabilis*	In vitro-agar well diffusion assay, MIC and MBC determination	Honey showed significant antibacterial activity at 100% and 75%, weaker at 50%. MIC: 1.57–6.25 mg/mL, MBC: 3.13–12.5 mg/mL. Suggests potential for treating wound infections.	[153]
Ethiopian honeys—*Apis mellifera* (white and yellow), stingless bee honey	*Staphylococcus aureus* ATCC 25923, *Escherichia coli* ATCC 25922, MRSA, resistant *E. coli*, *Klebsiella pneumoniae* (R)	In vitro-agar diffusion (Mueller Hinton), broth culture, MIC and MBC determination	Stingless bee honey had highest inhibition (22.27 mm) and lowest MIC (6.25%). All honeys bactericidal at MBC = 12.5 mg/mL. Resistant strains were susceptible to honey.	[154]
Four local honeys (“honey-2”)	MRSA clinical isolates from wound infections	In vitro-disc diffusion, broth dilution (MIC, MBC)	All honeys active against MRSA. “Honey-2” was most effective (MIC/MBC 9.38–37.5%). Honey showed both bacteriostatic and bactericidal activity.	[155]
Rape honey enriched with *Rubus* spp. fruits (1%, 4%) and leaves (0.5%, 1%)	*Staphylococcus aureus* (planktonic and biofilm), *Escherichia coli*, bacteriophage phi 6 (viral surrogate)	In vitro-antibacterial (including biofilm), antiviral (phage test), antioxidant profiling (HPTLC, HPLC)	*Rubus*-enriched honey showed higher antioxidant, antibacterial and antiviral activity than plain rape honey. Best effects for 4% raspberry fruit and 1% blackberry leaf. Strong inhibition of *S. aureus* biofilm.	[156]

**Table 4:** Selection of articles on the antiviral effect of honey.

Material	Microorganism	Assay	Key Results	Reference
Rape honey + Rubus fruits/leaves	Bacteriophage phi6	Double agar overlay plaque assay	4% raspberry addition: ≥7.4 log_1__0_ PFU/mL reduction after 24 h.	[231]
Manuka honey	HIV-1 (RT)	Colorimetric HIV-1 RT inhibition assay	IC_5__0_ ≈ 14.8 mg/mL; inhibition due to MGO and 2-MBA.	[232]
Tualang honey	Chikungunya virus (CHIKV)	In vitro XTT + plaque assays (pre/post/virucidal)	Up to 99.71% inhibition via multiple mechanisms.	[233]
Chestnut honey	SARS-CoV-2 (COVID-19)	Questionnaire-based survey (n = 177)	No significant link between use and COVID-19 outcome.	[234]
Tilia amurensis honey	Influenza A virus (IAV)	In vitro macrophage assays (cytokines, IFN signaling)	Inhibited replication via IFN-1 and IFITM3 activation.	[235]
Castanea crenata honey	Influenza A virus (IAV)	In vitro + in vivo (mice): viral load, survival, inflammation	↑ survival by 60%, ↓ virus/inflammation, RIG-I/MAVS activation.	[236]
Korean Chestnut Honey (KCH)	*Herpes simplex virus* 1 (HSV-1)	In vitro: host cell assays, cytokines, inflammasome	Inhibited HSV-1 binding/replication, ↓ ROS, NF-κB, NLRP3.	[237]
Honey + Acyclovir	*Herpes simplex virus* 1 (HSV-1)	Systematic review of clinical trials	Weak evidence of improved healing with combination therapy.	[238]
Honey + Chokeberry fruit	Bacteriophage phi6	In vitro plaque assay; in vivo yeast oxidative stress test	↑ antiviral and antioxidant effect; 1–4% chokeberry effective.	[239]
Various honey samples	Norovirus VLPs (GII.4, GII.10)	HBGA binding inhibition assay; DLS; electron microscopy	Several honeys showed weak HBGA binding inhibition; stronger effect seen with propolis and date syrup.	[240]
Hovenia dulcis honey (HDH)	Influenza A virus (IAV)	In vitro: RAW 264.7 murine macrophages (viral proteins, IFN, ROS)	HDH inhibited viral replication, enhanced IFN-β via cGAS-STING-STAT1/2 pathway, reduced ROS via Sirt3/SOD2 upregulation.	[241]

↓: 60% increase in survival rate, reduction in virus/inflammation, activation of RIG-I/MAVS; ↑: increased antiviral and antioxidant activity; 1–4% aronia effective.

**Table 5:** Selection of articles on the antiviral effect of propolis.

Material	Microorganism	Assay	Key Results	Reference
Brown (Mexico), green and red (Brazil) propolis extracts; quercetin, caffeic acid, rutin	HCoV-229E	In vitro infection of MRC-5 lung fibroblast cells; cytotoxicity and antiviral activity (EC_5__0_, TC_5__0_)	All samples showed antiviral activity; green and brown propolis and quercetin had best EC_5__0_ values (19.08, 11.24, 77.21 µg/mL, respectively)	[251]
Bulgarian propolis extracts (6 samples)	HCoV OC-43, HRSV-2, HSV-1, HRV-14, HadV-5	In vitro CPE inhibition assay; virucidal activity; adsorption inhibition; cell protection tests	Strongest antiviral effect observed against HCoV OC-43; greater effect on enveloped viruses; significant inhibition of HSV-1 and partial inhibition of coronavirus adsorption; some extracts showed protective effect on host cells when applied pre-infection	[246]
Brazilian red and green propolis extracts (conventional and ultrasound-assisted extraction)	Bacteriophages MS2 and Av-08	In vitro assay measuring plaque-forming unit (PFU) reduction	Red and green propolis reduced MS2 and Av-08 titers by ~3 and ~4.5 Log_1__0_ PFU/mL, respectively; red propolis more effective; ultrasound-assisted extraction enhanced activity	[252]
Propolis, chitosan nanoparticles, propolis–chitosan mixture	Newcastle disease virus (NDV) isolates MW881875 and MW881876	In vitro TCID_5__0_ assay on Vero cells; cytotoxicity evaluation	All tested materials showed antiviral activity; propolis at 13 µg/mL reduced viral titer by 2.66 log_1__0_; propolis–chitosan mixture reduced by 2.5 log_1__0_; cytotoxic concentrations determined for each	[253]
Standardized hydroalcoholic extract of Eurasian poplar-type propolis (sHEP); caffeic acid phenethyl ester, galangin, pinocembrin	SARS-CoV-2	In vitro infection of VERO E6 and CALU3 cells; RNA quantification; microscopy; viral titration	sHEP reduced replication, cytopathic effects, and viral RNA levels; effects seen mainly in CALU3 cells; combination of three major components showed similar antiviral activity; pre-treatment protected cells but did not block viral entry	[254]
Nutraceutical formula (Solution-3) containing propolis, *Verbascum thapsus*, *Thymus vulgaris*, and polyphosphates	SARS-CoV-2 (EG.5), Influenza A (FLU-A), RSV-A	In vitro infection assays (qPCR); MIC/MBC testing; transcriptomic and ELISA analysis	Exhibited antiviral, antibacterial, and antifungal activity; enhanced innate immune responses via modulation of cytokines, chemokines, antimicrobial peptides, and complement; potential as prophylactic agent against viral and polymicrobial infections, including co-infections in COVID-19 patients	[255]
Ethanolic Anatolian propolis extracts (Pazar, Ardahan, Uzungöl)	Herpes simplex virus type 1 (HSV-1)	In vitro MTT assay, qRT-PCR, plaque reduction test; HPLC-UV phenolic profiling	All samples showed antiviral activity; higher phenolic content correlated with stronger inhibition of HSV-1; total phenolics ranged 44.12–166.91 mg GAE/g; flavonoids 12.50–41.58 mg QUE/g	[256]
Syrup containing propolis (450 mg/10 mL) and *Hyoscyamus niger* extract (1.6 mg/10 mL)	SARS-CoV-2 (COVID-19 patients)	Randomized clinical trial (n = 50); symptom monitoring over 6 days	Significant improvement of COVID-19 symptoms (e.g., cough, sore throat, chest pain, fever) vs. placebo; no effect on nausea or vomiting; suggests therapeutic benefit in mild-to-moderate cases	[257]
Aqueous extract and purified fractions of propolis from *Scaptotrigona aff. postica*	Zika virus, Chikungunya virus, Mayaro virus	In vitro infection of VERO cells; focus reduction assay; HPLC and mass spectrometry characterization	Crude extract reduced Zika (64×), Mayaro (128×), and Chikungunya (256× at 5% v/v); purified compound reduced Zika (16×), Mayaro (32×), Chikungunya (512×); strongest effect when added 2 h post-infection; antiviral effect was concentration dependent	[258]
Ethanol extract of propolis (EEP) encapsulated in PLGA–chitosan nanoparticles (EEP-NPs)	Herpes simplex virus type 2 (HSV-2)	In vitro Vero cell assay; cytotoxicity; gene expression analysis (ICP4, ICP27, gB)	EEP-NPs had low cytotoxicity and strong antiviral activity; inactivated viral particles; inhibited entry and release; reduced HSV-2 replication gene expression; showed sustained release profile	[259]
Propolis flavonoid ethanolic extract (PF); ferulic acid	Porcine parvovirus (PPV)	UPLC-QTOF-MS; in vitro antiviral screening; in vivo vaccine adjuvant test in sows	PF showed anti-PPV activity; ferulic acid identified as active component; PF enhanced vaccine-induced humoral (IgM, IgG) and cellular responses (IL-2, IL-4, IFN-γ) in sows; strongest adjuvant effect on Th1/Th2 responses and lymphocyte proliferation	[260]
Liposomal formulation of Egyptian propolis (optimized via response surface methodology); rutin; CAPE	SARS-CoV-2	Molecular docking (3CL protease, spike S1); IC_5__0_ assay; RT-PCR for viral replication inhibition	Liposomes enhanced antiviral activity vs. crude extract; IC_5__0_ of liposomes = 1.183 µg/mL vs. 2.452 µg/mL for extract (*p* < 0.001); liposomal propolis significantly inhibited SARS-CoV-2 replication; effect comparable to remdesivir (*p* < 0.0001)	[261]

**Table 6:** Selection of articles on the antiviral effect of bee venom.

Material	Microorganism	Assay	Key Results	Reference
Bee ventom (BV)	MERS-CoV (Middle East respiratory syndrome coronavirus)	Cytopathic effect (CPE) inhibition assay	Crude BV showed mild anti-MERS-CoV activity (SI = 4.6)	[218]
Bee venom (BV), melittin (MLT)	Influenza A (H1N1), VSV, RSV, HSV, Enterovirus-71, Coxsackievirus (H3)	In vitro viral replication inhibition; in vivo mouse protection assay	BV and MLT inhibited replication of enveloped and non-enveloped viruses; MLT protected mice against lethal Influenza A	[262]
Bee venom (BV)	Adenovirus-7 (DNA virus), Rift Valley fever virus—RVFV (RNA virus)	End point calculation assay (virus depletion titer)	BV showed strong antiviral activity against Adeno-7 (1.66 log_1__0_/mL) and RVFV (3.34 log_1__0_/mL), superior to interferon	[267]
Bee venom (BV)	Human papillomavirus (HPV-16, HPV-18)	In vitro and in vivo assays (cell proliferation, mRNA/protein expression)	BV downregulated HPV16 E6/E7 expression and suppressed growth of HPV16-infected CaSki and TC-1 cells; weaker effect on HPV18-infected HeLa cells	[268]
Bee venom (BV)	SARS-CoV-2	Immunodiagnostic antigen titer reduction; plaque reduction assay	BV showed antiviral activity (EC90 = 2.23 mg/mL); less potent than wasp venom	[269]
Bee venom (BV)	Hepatitis C virus (HCV, genotype 2a)	In vitro cell culture (Huh7it-1 cells, JFH1 strain); IC50 and CC50 determination	BV inhibited HCV entry with IC50 = 0.05 ng/mL; CC50 = 20,000 ng/mL; no effect from major components (melittin, apamin, MCD); antiviral action likely from minor venom components	[263]
Bee venom sPLA_2_	HIV-1 (macrophage- and T cell-tropic strains)	HIV-1 replication inhibition in human leukocytes; intracellular capsid release	sPLA_2_ from bee venom inhibited HIV-1 replication (ID_5__0_ < 1 nM); effect independent of enzymatic activity; involved specific binding to host cells	[270]

## Data Availability

Not applicable.
